# QAAPT: an interoperable web-based open-source tool for antimicrobial resistance data analysis and visualisation

**DOI:** 10.3389/fmicb.2025.1513454

**Published:** 2025-02-07

**Authors:** Mohammad Julhas Sujan, Sanjay Gautam, Ahmed Taha Aboushady, Adam Clark, Sooyoung Kwon, Hea Sun Joh, Marianne Holm, John Stelling, Florian Marks, Nimesh Poudyal

**Affiliations:** ^1^Department of Antimocrobial Resistance, International Vaccine Institute, Seoul, Republic of Korea; ^2^Brigham and Women's Hospital, Harvard Medical School, Boston, MA, United States; ^3^Cambridge Institute of Therapeutic Immunology and Infectious Disease, University of Cambridge School of Clinical Medicine, Cambridge, United Kingdom; ^4^Heidelberg Institute of Global Health, University of Heidelberg, Heidelberg, Germany; ^5^Madagascar Institute for Vaccine Research, University of Antananarivo, Antananarivo, Madagascar

**Keywords:** antimicrobial resistance, data analysis, data visualisation, surveillance, low-middle-income countries

## Abstract

The analysis and visualisation of antimicrobial resistance (AMR) surveillance data is a crucial challenge, especially in high-burden, low-middle-income countries. We describe the design, development, integration, and implementation of the Quick Analysis of Antimicrobial Patterns and Trends (QAAPT) tool for AMR data analysis and visualisation. The QAAPT tool was created by the Capturing Data on Antimicrobial Resistance Patterns and Trends in Use in Regions of Asia project, led by the International Vaccine Institute (IVI). This open-source web-based tool/application generates statistical and visual outputs of AMR data, offers data curation options, and can be integrated with laboratory information management systems. The QAAPT tool is user-friendly and is operable by someone with limited expertise in software or programming. As a part of the project, the tool was used to analyse data from 72 laboratories across 7 Asian countries. In this study, we present the technical aspects of tool development and highlight implementation outcomes for analysing and generating visual reports from more than 2.37 million highly heterogeneous antimicrobial susceptibility test data points.

## Introduction

Antimicrobial resistance (AMR) is considered a significant global public health threat, often referred to as a ‘silent pandemic’ (World Health Organization, 2023). To effectively combat the emergence and spread of AMR, it is crucial to establish a robust surveillance system that provides a comprehensive understanding of the burden and informs policy on intervention and control strategies (National Academies of Sciences, 2021). However, in low-and middle-income countries (LMICs), which bear a disproportionately high burden of bacterial drug resistance, there are challenges accessing user-friendly and reliable tools for AMR data collection, analysis, and visualisation (Sharma et al., 2022). Whilst open-source platforms such as WHONET,^1^ R package for AMR,^2^ and AMRcloud^3^ already exist, they often have limitations such as technical requirements, cost, usability with diverse datasets, and integration options with existing data management systems.

The Capturing Data on Antimicrobial Resistance Patterns and Trends in Use in Regions of Asia (CAPTURA) project, funded by a Fleming Fund Regional Grant and led by the International Vaccine Institute (IVI), aimed to significantly increase the volume of available AMR, antimicrobial consumption, and antimicrobial use data for informed decision-making. In collaboration with local governments and private and public healthcare facilities, this initiative unearthed, collated, and analysed AMR data to provide insights at local, regional, and interregional levels (Holm et al., 2023). Informed by CAPTURA phase 1 (2019–2023), scoping reports from 12 countries, a secure centralised data repository, a data warehouse, and a visualisation tool were created. Initially, when data were collected from the laboratories, WHONET software was used to unify, curate, and analyse the data. This was followed by developing a QAAPT data analysis and visualisation tool to prepare detailed reports at both country and facility levels. Data contributors from 72 laboratories could log into the platform to download their raw and cleaned data, access detailed reports, and use the dashboard with predefined and customisable filters (Poudyal et al., 2023). In this study, we describe QAAPT’s design, development, implementation, interoperability, features, and lessons learned from its rollover in seven South and Southeast Asian countries.

## Methods

### Application development

The QAAPT application was developed using a free and open-source programming language, the Laravel framework, and the MySQL database management system. Laravel is a free and open-source Hypertext Preprocessor (PHP) framework (Laravel, 2020) that includes an Object Relational Mapper (ORM) called Eloquent and built-in mechanisms for constructing database migrations and seeders (PHP, 2021). Laravel’s authentication incorporates “guards” and “providers.” Guards define the way users are authenticated for each request. The QAAPT tool was built to provide an easy and secure user authentication process using this strategy. In addition, JavaScript packages such as HighCharts and AmChart were included for data visualisation. The user interface was designed using Hypertext Markup Language (HTML) and Cascading Style Sheets (CSSs).

**Figure 1:**
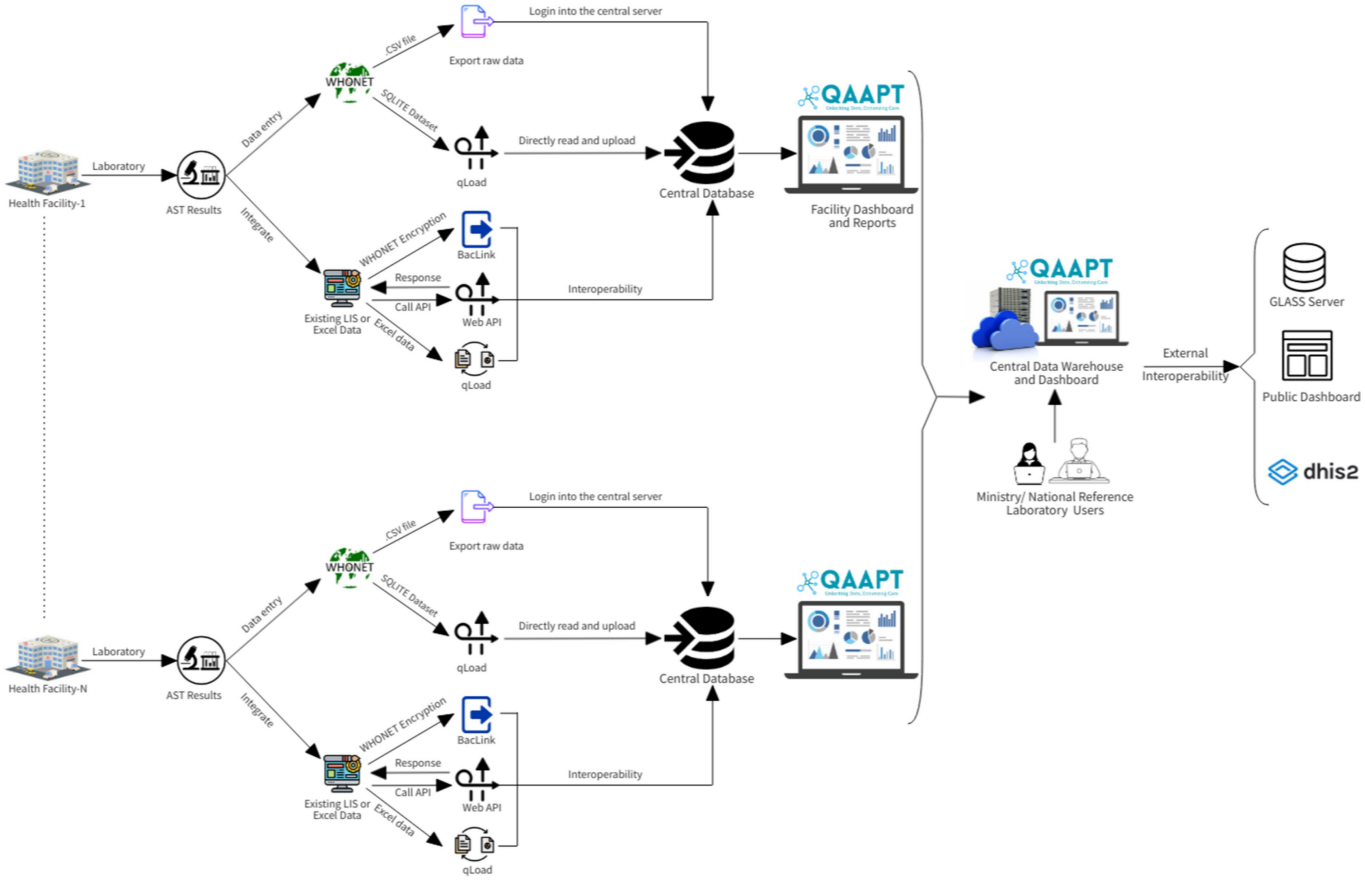
QAAPT workflow shows the steps/phases of data capture, analysis, and integration into national surveillance systems and external platforms such as the GLASS server.

**Figure 2:**
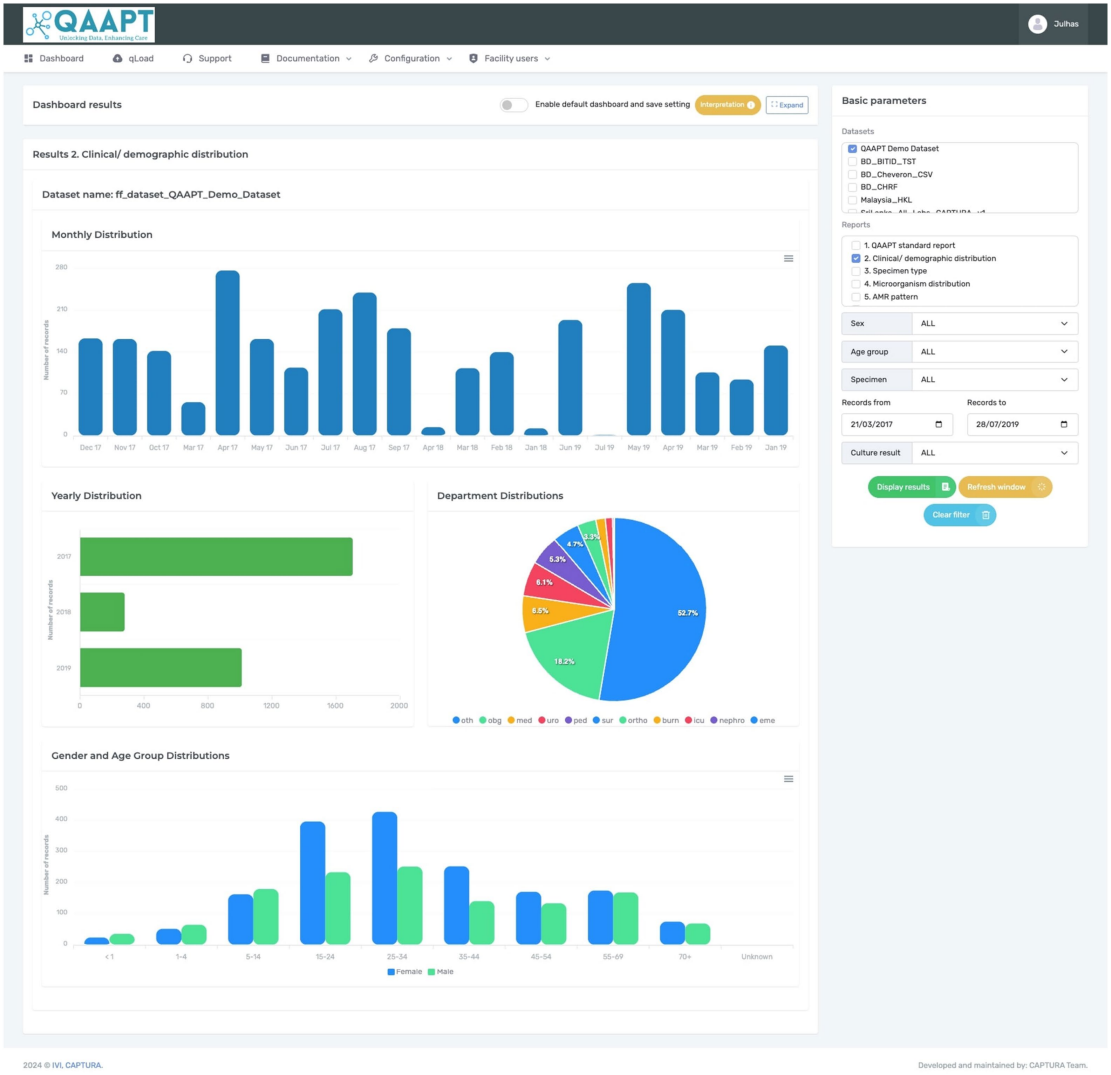
Typical QAAPT report showing one of the outputs (clinical and demographic distribution) of analysed data.

For the QAAPT, source code collaboration and backup were managed using the Bitbucket web-based version control system (Bitbucket, 2021). This public repository supports forking and making changes to fit users’ needs. A cloud-based hosting solution was created for the development environment and production server. However, personal computers were used for development and programming; thus, any modifications were committed (pushed) to the distant ‘origin’ repository, and other developers pulled (retrieved) the new version (Figures 1, 2).

Using the web Application Programming Interface (API), the QAAPT tool is designed to integrate into the District Health Information System (DHIS2) for advanced visualisation and store data as a national repository (Asaduzzaman et al., 2024). In close collaboration with microbiologists, physicians, epidemiologists, public health practitioners, and biostatisticians, the QAAPT tool is meticulously developed to address healthcare professionals’ diverse needs and improve AMR management. Following its development, QAAPT is continuously reviewed by senior managers and software engineers from the WHONET team to ensure its robustness and reliability. Expert feedback and comprehensive evaluations are ongoing processes to refine and enhance the tool’s functionality.

### Application launch and validation

To launch the application, users must register to obtain their login credentials. Since not all the modules in this application are available to all users, the registration process requires users to define their role to tailor access to appropriate resources. A super-admin assigned to an organisation can create multiple roles and assign users. All the essential packages and extensions are installed during the initial configuration. After successfully logging in, the user can access two main areas: the dashboard, which allows dataset selection and populates all predefined descriptive statistics, and the top navigation, which allows users to retrieve, import, curate, and visualise trends, patterns, and correlations. The right panel enables users to apply different filters. Users can import output files from WHONET SQLite data files, text files, and Excel files from the majority of laboratory information systems. The QAAPT tool also allows integration with any live database supporting web API interfaces. The data importation process encrypts patient personal identifiers such as patient ID, name, and date of birth. The tool requires at least six mandatory variables (patient ID, age, sex, specimen type, specimen collection date, and microorganism type) in the dataset for successful importation and downstream analysis.

The QAAPT application was implemented to analyse and visualise the data collected from 72 laboratories across 7 countries, namely Bangladesh, Bhutan, Laos, Nepal, Papua New Guinea, Timor-Leste, and Sri Lanka (Poudyal et al., 2023). The analysis of antimicrobial susceptibility test records was based on Clinical and Laboratory Standards Institute (CLSI) recommendations (Clinical and Laboratory Standard Institutes, 2022). The internal logic computed the AST data for a bacterial isolate with at least 20 records and provided an option to set the minimal threshold to 30 (Clinical and Laboratory Standard Institutes, 2022). The WHONET breakpoint algorithms were adopted to convert the zone of inhibition or minimum inhibitory concentration (MIC)/E-test values to determine the percentage of resistance patterns, trends, correlations, and antibiograms (QAAPT, 2022). For quarterly and half-yearly AST trend analysis, the first isolates per patient per species are considered. The tool systematically identifies the first isolates per patient per species by selecting patients’ first visits with a unique patient registration number and species identifier (Luz, 2021; Berends, 2021).

## Results

### QAAPT tool

The QAAPT application is developed and integrated into the International Vaccine Institute’s cloud server. This tool is accessible on the web^4^ or can be readily installed and customised as an application on any physical dedicated server, local PC, or cloud. For the CAPTURA project, participating facilities used QAAPT as either a single-user or multi-user tool. Some facilities utilised it as a central data warehouse, enabling them to submit their datasets with access credentials. This setup also allowed central authorities, such as the Ministry of Health or the National Reference Laboratory, to access and visualise the data.

The QAAPT single-page dashboard with multiple-view architecture facilitates quick and efficient navigation across different functions. Users could select desired outputs, such as positive and negative reports on bacterial culture and distributions of culture results for selected day/month/year ranges, age, and sex, amongst others, using various filter options on the dashboard. The simple user interface allows for the export of reports in multiple forms, such as bar graphs, pie charts, line graphs, stacked bar and column graphs, and combinations of diagrams with outputs in various formats, including .xls, .csv, .pdf, .png, .jpeg, and .svg. QAAPT integrates metadata tables from WHONET for AST breakpoint/s, microorganism, and antibiotic data to ensure consistency and standardised output formatting (Figure 1).

### Analysis of bacterial culture

The CAPTURA project received highly heterogeneous data in different formats (such as .xls or. SQLITE format) and was successfully imported to QAAPT. To analyse and visualise data on bacterial culture, QAAPT categorised clinical specimen types into eight broad categories (blood, genital, respiratory, soft tissue and body fluids, stool, urine, other, and unknown). The QAAPT algorithm automatically groups common bacteria such as *Escherichia coli*, *Klebsiella* spp., *Enterococcus* spp., *Acinetobacter* spp., *Staphylococcus* spp., *Staphylococcus aureus*, *Pseudomonas* spp., and *Proteus* spp. and specimens from the MS Excel file or WHONET output during the data importation process. Similarly, the tool detected and recoded results stored as unidentified species as unknown, no-growth as xxx, no significant growth as xsg, contamination as con, normal flora as nor, oral flora as ora, vaginal flora as vag, mixed bacterial species as mix, and enteric pathogens as xep. The empty cells were replaced with ‘NULL’ values. Outputs were available by specimen type, specimens with positive or negative culture reports, and options for selected clinical and demographic variables such as location, age, and sex. A summary of the results for all samples, including the total records, total numbers of positive and negative bacterial cultures, and differentiation between true pathogens, mixed growth, contamination, and normal flora (such as oral and vaginal flora), can be extracted along with the classification of missing records for organisms and specimens. Similarly, the tool allows the visualisation of most reported microorganisms and categorises Gram-positive and Gram-negative bacteria (Figure 2).

### Analysis of antimicrobial susceptibility

The QAAPT tool incorporates the CLSI and the European Committee on Antimicrobial Susceptibility Testing (EUCAST) guidelines from 2012 to 2024, allowing users to select specific guidelines to analyse retrospective data. Using the right sidebar in the dashboard, users can choose an imported dataset to analyse data on AMR. By default, the application shows AST data for the 25 most common pathogens (in the dataset) for all the available antibiotics. The algorithm adopts WHONET antibiotic codes to be tested against a particular pathogen. The antibiotic susceptibility test results can be visualised by types of specimens, bacteria, antibiotics tested, antibiotic test method, and interpretation methods, and they are classified into three categories: susceptible, resistant, and intermediate. The results can be customised for selected microorganisms or clinical demographic variables. The result is displayed as bar graphs, pie charts, heat maps, or in a tabulated format with values for frequency and percentage.

The antibiogram panel enables users to generate cross-tabulations by selecting various variables and time periods, allowing for the visualisation of cumulative antibiogram data as percentages, complete with numerators and denominators. In addition, users can export antibiogram results in Excel and HTML formats. The panel also provides various types of antibiogram results, including the distribution of bacterial growth in different specimens, the patterns of organisms isolated, and the distribution of microorganisms by ward (inpatient and outpatient wards), sex, and specimen type and AST trends and patterns of significant Gram-positive and Gram-negative organisms.

Similarly, the QAAPT tool allows users to generate comprehensive reports on multi-drug resistance (MDR) for an individual microorganism. Users can analyse and prepare reports on MDR prevalence per pathogen, specimen types, and demographic categories, enabling a detailed examination of antibiotic resistance trends and patterns. Users can select the desired dataset to generate an MDR report and apply relevant filters, such as organism type, specimen source, or patient demographics. The tool automatically identifies MDR strains based on predefined criteria, such as resistance to three or more classes of antimicrobial agents. This feature allows for the consistent and standardised reporting of MDR across different settings, facilitating comparability and benchmarking.

### Performance of large dataset imports and data security

A wide range of datasets (files between 450,000 and 1.1 million patient records) were successfully tested. On average, it took approximately 1 min to import and process data containing 10,000 unique records (approximate file size: 50 megabytes). The cloud server security protocols and instructions for configuring the droplet servers were adopted for additional security. Similarly, the QAAPT tool used the Laravel permission package to limit access to specific menus, modules, and datasets, allowing users to view only selected results they are authorised to access. A data privacy policy and terms of use guidelines have been developed and are available in the laboratory registration section. The policy outlines the information collected, its use, the data-sharing strategy, security measures, data retention practices, and user rights. It also specifies that the system does not collect personal identifiers, further enhancing data security.

### Interoperability with DHIS2

In collaboration with one of the participating government agencies, we designed a feature using the DHIS2 web API to generate the event programme’s metadata and data for individual isolates. Following a successful dry run with a test dataset, the dashboard and outputs were created for other AMR data users at the national and sub-national levels to visualise it on DHIS2.

### User feedback

The implementation of QAAPT has garnered significant positive feedback from various healthcare professionals who have integrated the tool into their daily operations. Their experiences highlight the tool’s impact on improving diagnostic accuracy, the efficiency of data management, and overall healthcare delivery. Feedback collected post-implementation of the tool reflects the tool’s ability to simplify data handling, streamline decision-making, and facilitate the sharing of critical information. Its user-friendly interface and robust export features have proven to be significant assets, allowing for improved management of patient treatment and adherence to high standards in laboratory operations.

## Discussion

We developed a web-based tool, QAAPT, and implemented it for the CAPTURA project to analyse and visualise the AMR data collected from 72 healthcare facilities in 7 Asian countries. All the information, including account details and AMR data, was stored on a cloud-based platform hosted by IVI. The tool incorporates secure encryption protocols such as secure shell (SSH), firewalls, virtual private clouds, and transport layer security (TLS) encryption, making the tool a safe option for public health data analysis. According to the data transfer agreement (DTA) with participating laboratories, all data stored on the QAAPT platform will be deleted from the central location at the end of the project.

The tool offers a user-friendly interface in settings with basic computer and Internet infrastructure. The tool’s ease of use enables rapid training for new staff, which is especially advantageous in environments with high staff turnover and limited training resources. The QAAPT function to import datasets in different formats and interoperability with DHIS2 through a web API makes it possible to access data from multiple sources to a central location (such as the ministries) for decision-making and early detection of the emergence and spread of bacterial infections/drug-resistant infections.

Initially, there were loading issues when handling one million records on the dashboard. Based on this experience, lazy-loading technology was implemented in the dashboard. Lazy loading delays the loading or initialisation of resources or objects until needed, aiming to improve performance and conserve system resources (Olawanle, 2023). In addition, its multi-layered user management system allows simultaneous access for laboratory managers, personnel, policymakers, and project managers. Integration capabilities extend to diverse hospital and laboratory solutions, identification methods, and antimicrobial susceptibility testing (AST) performance across all laboratories, facilitating the establishment of a robust AMR surveillance system.

Currently, several commercial platforms are available, such as AMRMap and ATLAS, to manage and analyse AMR data. However, a common barrier to their adoption in resource-limited settings is associated with cost, licence requirements, proprietary source code, reliability, interoperability with existing platforms, and technical requirements (instruments and human resources) (World Health Organization, 2015). QAAPT offers seamless interoperability, open-source availability, and a straightforward dashboard, accommodating users with diverse technical backgrounds. This led to the successful implementation of QAAPT to analyse and visualise diverse datasets in different countries. The initial version of the tool presented here had several limitations. For instance, it did not include historical guidelines that could aid in retrospective data analysis and interpretation of past events. In addition, there was no classification of antibiotics into different groups. Furthermore, the current evaluation of the tool did not gather any qualitative or quantitative data to compare it with existing platforms. Instead, this study concentrated on aspects of tool implementation and user perception. The IVI-led CAPTURA project used QAAPT to manage and disseminate data from Phase I (2019–2023) (Sujan et al., 2023) and plans to launch an updated version for CAPTURA Phase II, featuring enhanced features and improvements. It is essential to keep developing the tool and expanding its usage to various locations. However, a significant challenge remains in sustaining the tool beyond the project’s initial scope and duration. We advocate for a collaborative effort amongst diverse stakeholders in the field of AMR to create opportunities for the continued development of emerging and promising tools such as QAAPT.

## Data Availability

The original contributions presented in the study are included in the article/supplementary material, further inquiries can be directed to the corresponding author.

## Footnotes

^1^ https://whonet.org/

^2^ https://msberends.github.io/AMR

^3^ https://amrcloud.net/en

^4^ https://qaapt.com/
